# High Efficacy of Rose Bengal in Reducing the Pathogenicity of *Escherichia coli* Isolated From Diarrheal Infections

**DOI:** 10.1155/ijm/4912438

**Published:** 2025-06-18

**Authors:** Christ Dieuveil Bayakissa Malanda, Christian Aimé Kayath, Nicole Prisca Makaya Dangui Nieko, Frédéric Yannick Okouakoua, Ndelani Nkalla Lambi, Dieuvit Haïdide Kibamba Niangui, Sergy Patrick Junior Bissoko, Duchel Jeandevi Kinouani Kinavouidi

**Affiliations:** ^1^Laboratory of Cell and Molecular Biology (BCM), Faculty of Science and Technology, Marien Ngouabi University, Brazzaville, Congo; ^2^Higher Teacher Training School (ENS), Marien Ngouabi University, Brazzaville, Congo

## Abstract

Diarrheal infections, a leading cause of global morbidity and mortality, are frequently attributed to pathogenic *Escherichia coli* strains. The rise of antibiotic resistance among these pathogens necessitates the exploration of alternative therapeutic agents. This study is aimed at evaluating the Rose Bengal effect to fight antibiotic resistance in pathogenic *E. coli*. Using a combination of in vitro assays—including microbiological isolation, 16S RNA molecular identification, acid resistance testing, biofilm and swarming assays, hemolytic activity evaluation, and antibiograms—and in vivo analysis with *Rhynchophorus phoenicis* larvae, 22 *E. coli* isolates were obtained. Molecular analyses identified four pathogenic strains: KNH8 (PQ864811), KNH11 (PQ864812), KNH14 (PQ864813), and KNH16 (PQ864814), classified as enterotoxigenic *E. coli* (ETEC) and enteropathogenic *E. coli* (EPEC). Pathogenicity assessments revealed that Rose Bengal (200 *μ*M) significantly reduced acid and bile salt resistance, biofilm formation, swarming motility, and hemolytic activity in all strains. Furthermore, Rose Bengal enhanced the sensitivity of these strains to five antibiotics—imipenem, kanamycin, chloramphenicol, gentamicin, and amoxicillin/clavulanic acid (AMC)—with increases ranging from twofold to sixfold in pathogenic strains (KNH8, KNH11, KNH14, and KNH16). These effects were further corroborated by in vivo testing using *R. phoenicis* larvae. The findings highlight the virulent potential of these *E. coli* strains and suggest Rose Bengal as a promising antimicrobial agent against multidrug-resistant pathogens.

## 1. Introduction

Microbial infections have long been a leading cause of human pathologies. Among the microorganisms involved in these pathologies, *Escherichia coli* holds a prominent place [1]. *E. coli* is a bacterium belonging to the class of *γ*-proteobacteria and the family Enterobacteriaceae. Commonly found in the colon of animals, where it plays roles such as aiding digestion, *E. coli* is now classified among the bacteria that are of increasing medical interest [2]. While some strains of *E. coli* are commensals, enteropathogenic, enteroinvasive, enterohemorrhagic, and enteroaggregative strains are highly virulent and responsible for numerous infections, including diarrhea and foodborne infections [3]. In developing countries, diarrheagenic *E. coli* accounts for 30%–40% of acute diarrhea cases in children under the age of 5 [3]. The World Health Organization (WHO) reports that *E. coli* strains cause millions of diarrheal disease cases annually, particularly affecting young children and individuals with weakened immune systems [4].

Enterotoxigenic *E. coli* (ETEC) and enteropathogenic *E. coli* (EPEC) represent a major public health concern worldwide, particularly in developing countries, where they are responsible for an alarming number of diarrhea cases [3, 5, 6]. Each year, ETEC is responsible for approximately 200 million cases of diarrheal disease and around 100,000 deaths, primarily among young children, making it a significant contributor to global morbidity and mortality [7].

Infections caused by ETEC are primarily attributed to two main virulence factors: toxins and adhesion structures. ETEC strains produce specific enterotoxins that promote colonization of the small intestine and induce diarrhea by stimulating intestinal epithelial cells to secrete excessive fluid. Additionally, these bacteria possess filamentous structures known as F41 pili, which enable them to adhere to intestinal cells, thereby initiating the infectious process [8].

In the Republic of Congo, particularly in Dolisie (Niari Department), a gastroenteritis outbreak was declared by the Congolese government, primarily involving enterobacteria such as *E. coli*, *Shigella*, and *Salmonella* species. This outbreak has been linked to over 100 reported deaths (data currently being published).

Given the alarming nature of this issue, understanding bacterial pathogenicity is essential for identifying effective therapeutic alternatives. This study contributes to the search for solutions tailored to local public health challenges while also addressing a global concern: antimicrobial resistance.

This resistance is further aggravated by the emergence of extended-spectrum *β*-lactamase (ESBL)–producing strains, which can hydrolyze a broad spectrum of *β*-lactam antibiotics, thereby significantly limiting available treatment options. The identification of new therapeutic targets against these resistant bacteria represents a critical and timely challenge [9].

A previous study conducted in our laboratory demonstrated that Rose Bengal can inhibit the function of the SecYEG protein export system by specifically blocking the SecA ATPase complex [10]. The SecYEG system is a vital mechanism for bacterial survival and plays a key role in the expression of virulence by directly or indirectly facilitating the secretion of virulence effectors [11]. In this context, exploring Rose Bengal as an innovative approach to attenuate the pathogenicity of *E. coli* strains could represent a promising therapeutic avenue. Within this framework, we proposed to evaluate the effect of Rose Bengal on the pathogenicity of *E. coli* strains in order to assess its potential as an alternative therapeutic strategy.

## 2. Methods

### 2.1. Biological Tools and Collection

This study was carried out over a 6-month period, from March to September 2024, at the Laboratory of Applied Microbiology and Molecular Biology of the National Institute for Research in Exact and Natural Sciences (IRSEN) located in Brazzaville. The biological material used included stool samples—provided by the bacteriology laboratories of the Pierre Mobengo Central Military Hospital and Talangaï Hospital—as well as larvae of *Rhynchophorus phoenicis*, commonly known as “Nsombé,” purchased from the municipal market in the Bacongo district (Arrondissement 2), Brazzaville. Samples were collected in sterile containers, placed in coolers with ice packs, and transported to the IRSEN Laboratory for further analysis.

### 2.2. Isolation, Identification, and Characterization

One gram of stool was placed in a tube containing 9 mL of sterile saline solution, and the mixture was homogenized using a vortex to create the stock solution. A series of decimal dilutions (10^−1^, 10^−2^,…) was then performed from the stock solutions obtained for each sample. The inocula from each sample were plated onto eosin and methylene blue (EMB) agar. The Petri dishes were then incubated in aerobic conditions at 37°C for 24 h. After growth, the colonies were subcultured onto the same medium to confirm the purity of the strains. Only pure strains were selected and used for the subsequent steps. To ensure purity, the colonies were subcultured three times until identical colonies were obtained. Phenotypic characterization of the strains was based on their morphological and biochemical characteristics. A macroscopic examination of the isolated colonies was done. This included evaluating parameters such as shape, color, consistency, and opacity. The 3% KOH test was used to determine whether the bacteria were Gram-negative or Gram-positive. The catalase test had been done as previously demonstrated [12]. Analytical Profile Index (API) 20E Test was performed to identify *E. coli* strains as previously demonstrated by the procedure manufacturer.

The Voges–Proskauer test had been performed. The MR-VP broth was prepared by dissolving 7 g of peptone, 5 g of fructose, and 5 g of disodium phosphate (Na_2_HPO_4_) in 100 mL of distilled water, followed by sterilization at 121°C for 15 min. For the Voges–Proskauer test, 1 mL of this sterile broth was transferred to a test tube, and 50 *μ*L of bacterial suspension was added. The mixture was incubated at 37°C for 24 h. After incubation, 3–6 drops of 5% *α*-naphthol and 2–3 drops of 40% KOH were added, with thorough mixing after each addition. The tube was then allowed to stand for 15–30 min before interpreting the results: a red ring indicated acetoin production by the bacterium, while its absence indicated no acetoin production. Pure 24-h cultures were stored in Eppendorf tubes containing BHI supplemented with 30% glycerol at −20°C for further analysis.

Finally, for each strain, hemolytic activity detection was performed. Blood agar had been prepared by adding 5% blood and then inoculating our bacterial isolates onto the surface of the agar by streaking. The Petri dishes were then incubated at 37°C for 24 h.

### 2.3. Genomic DNA Extraction, Molecular Identification, and Bioinformatics Analysis

Isolates (KNH8, KNH11, KNH14, and KNH16) were subjected to genomic DNA extraction and purification. This experiment was carried out using the NucleoSpin Microbial DNA Kit (Macherey-Nagel, Germany). Briefly, isolates were cultured in 5 mL of LB broth for 24 h at 37°C with shaking. DNA purity was assessed by the UV absorbance ratio (260/280 nm). One microliter of template DNA with concentrations equalling 10–20 ng/*μ*L was used. Universal 16S rRNA primers fD1 (5⁣′-AGAGTTTGATCCTGGCTCAG-3⁣′) and rP2 (5⁣′-ACGGCTACCTTGTTACGACTT-3⁣′) were used to amplify the 16S RNA gene (Eurogentec). Five microliters of each amplification product was mixed with 2 *μ*L of loading buffer (Bioke, the Netherlands). The mixtures were then electrophoresed on a 1% (*w*/*v*) agarose gel. The molecular weight marker used was the 10-kb 2-Log DNA sample (Bioke, the Netherlands). PCR products were purified using the Gel Extraction Kit (Omega Biotek), and the purified products were subjected to Sanger sequencing (3130xl Genetic Analyzer, Applied Biosystems). The sequences obtained were aligned with Bio Numerics 7.5 software (Applied Maths, Belgium) and manually corrected to resolve the discrepancies between the sense and antisense strands. The sequences were compared with homologous sequences contained in sequence databases through the NCBI portal using the BLASTn program (https://www.ncbi.nlm.nih.gov/).

### 2.4. Evaluation of Pathogenicity in *E. coli*

#### 2.4.1. Gastric pH Resistance Test

To assess the ability of the isolates to withstand gastric pH, a broth simulating gastric juice (pH = 2) was prepared. Each isolate was inoculated into this broth, and the optical density (OD) was measured at the initial time (T₀). The preparations were then plated onto TSA agar plates and incubated at 37°C for 24 hours. Afterward, the broths containing the same bacteria were incubated at 37°C for an additional 8 hours. Following this period, the OD was measured again, and a new inoculation was performed on TSA agar plates. These plates were incubated at 37°C for 24 hours, allowing the survival of the bacterial cells in an acidic environment to be evaluated.

#### 2.4.2. Bile Salt Resistance Test

The bacterial isolates were reactivated and the colonies were resuspended in 1 mL of physiological saline. Nutrient broth containing varying concentrations of bile salts (from 0.3% to 3%) was prepared. A standard broth without bile salts served as the control. A 100 *μ*L suspension of the bacterial sample was added to each tube, and the initial OD (*T*_0_) was recorded. Each mixture was then plated on TSA agar and incubated at 37°C for 24 h. The broth cultures were also incubated at 37°C for 8 h, after which a second OD measurement was taken. Samples were then inoculated onto fresh TSA agar plates and incubated again at 37°C for 24 h.

#### 2.4.3. Biofilm Formation Detection: A Qualitative and Quantitative Method

*E. coli* strains were cultured for 18 h and then inoculated onto EMB agar plates, which were incubated for 24 h. A single colony was suspended in 5 mL of sterile nutrient broth in test tubes and incubated for 24 h at 37°C under aerobic conditions. After incubation, the contents of the tubes were discarded, and the tubes were washed twice with 0.1 M phosphate-buffered saline (PBS) to remove planktonic cells. The remaining biofilm was stained with 2% crystal violet and incubated at 37°C for 20 min in an incubator (INCU-Line®, United Kingdom). The stained tubes were then rinsed with distilled water, and the adhered bacterial film was solubilized using 37% glacial acetic acid. The biofilm biomass was quantified by measuring absorbance at 550 nm using a spectrophotometer.

#### 2.4.4. Swarming Motility Detection

Swarming motility detection is a method used to assess the ability of certain bacteria to move collectively across a semisolid surface. To perform the test, 1 mL of an overnight bacterial culture grown in BHI at 37°C was transferred into 50 mL of fresh BHI medium. The culture was then incubated at 37°C with shaking at 250 rpm until it reached an OD between 0.6 and 0.7. Afterward, 10 *μ*L of the culture was spotted at the center of a Petri dish containing LB medium supplemented with 0.5% dextrose and 0.5% agar. The plates were incubated at 37°C, and the swarming behavior was assessed after 24 h. Only the positive and negative control strains were tested under these conditions.

#### 2.4.5. Antibiogram

Antibiotic susceptibility testing was carried out using the disk diffusion method on agar, following the guidelines of the French Society of Microbiology. Three to four well-isolated colonies of the test strain were transferred into 5 mL of physiological saline. After homogenization using a vortex, the bacterial suspension was adjusted to match the turbidity of a 0.5 McFarland standard [13]. The inoculum was evenly spread onto the surface of Mueller–Hinton agar using a sterile cotton swab. Each 90 mm Petri dish contained 25 mL of medium, ensuring a consistent agar thickness of 4 mm. The plates were allowed to dry for 10 min at room temperature. Antibiotic-impregnated disks were then placed equidistantly on the agar surface using sterile forceps. Plates were incubated at 37°C under aerobic conditions for 24 h. Inhibition zones were then measured, and results were interpreted according to the criteria set by the French Society of Microbiology.

### 2.5. In Vivo Evaluation of Pathogenicity Using the *R. phoenicis* (Nsombé) Animal Model

To evaluate the experimental infection model in *R. phoenicis* larvae, several parameters were assessed: larval survival up to 72 h postinoculation, proliferation of *E. coli* strains in the hemolymph following infection, and the immune response measured through melanization. This experiment was developed in the laboratory based on a previously published method [14].

To assess the virulence of *E. coli* strains, preliminary tests were conducted using bacterial doses ranging from 10^4^ to 10^9^ CFU/*μ*L. These tests, involving 24 larvae, allowed for the identification of strains with distinct virulence phenotypes and the determination of appropriate bacterial doses for subsequent experiments.

Briefly, *E. coli* strains were first cultured on EMB agar, then transferred to Mueller–Hinton broth, and incubated until reaching an OD of 0.5 at 600 nm. Cultures were then centrifuged, washed, and resuspended to a final concentration of 10^5^ CFU per larva. A volume of 10 *μ*L of each bacterial suspension was injected directly into the hemolymph of the larvae. Positive, negative, and PBS controls were included to validate the experimental results. To standardize conditions, larval feeding was suspended throughout the experiment.

The larvae were incubated at 37°C for 72 h in sterile glass jars, with daily monitoring to record mortality. At selected time points (2, 4, 6, 12, and 24 h), 20 *μ*L of hemolymph was collected, serially diluted, and plated on agar to count colony-forming units (CFUs), allowing the quantification of bacterial load in the hemolymph. The immune response was evaluated through visible melanization of the larvae, assessed macroscopically, and documented photographically using five larvae per condition for each experimental group.

### 2.6. Evaluation of the Effect of Rose Bengal on Pathogenic *E. coli* Strains

To evaluate the pathogenicity of the bacteria in the presence of Rose Bengal, a series of microdilution standards was prepared starting from an initial concentration of 0.3% to determine the ideal concentration capable of inhibiting the secretion system without killing the bacteria. The various dilutions prepared and tested were 500, 400, 300, 200, 100, 75, 50, 25, 15, and 52.5 *μ*M. Nutrient broths were then prepared with the aforementioned concentrations of Rose Bengal, and isolates were inoculated into the broths containing Rose Bengal before being incubated at 24°C for 24 h. To assess pathogenicity, the swarming motility test was chosen, following the same protocol described above. After incubation, the plates were examined; concentrations capable of inhibiting bacterial growth were discarded. Likewise, those that allowed growth but showed a profile very similar to the control (without Rose Bengal) were also excluded [10]. After determining the minimum concentration capable of inhibiting the secretion system (IC50) of Rose Bengal, all the previously conducted pathogenicity tests were repeated, following the same protocols. The various tests performed included the gastric pH resistance test, the bile salt resistance test, the biofilm formation test, the hemolytic test, and the swarming motility test, and the antibiogram have been conducted.

## 3. Results

### 3.1. Isolation, Characterization, and Molecular Identification

After sampling, isolation, purification, and subculturing, our study resulted in the identification of 54 isolates at the genus level, including 22 exhibiting cultural characteristics typical of bacteria of the *E. coli* genus. The isolation and purification medium used was EMB agar. We observed a phenotypic profile of the isolates, which appeared green with a metallic sheen characteristic of *E. coli* on EMB agar.

The strains isolated on EMB agar, following purification, were phenotypically characterized to confirm their belonging to the *E. coli* genus. The results are presented in the following table. Of the twenty-two isolates analyzed, 100% were found to be catalase-positive and KOH-positive, thus confirming their Gram-negative status. All these isolates demonstrated the ability to hydrolyze various tested substrates, including sorbitol, sucrose, maltose, lactose, galactose, and mannitol. However, none of the isolates could hydrolyze urea or produce acetone. These biochemical characteristics are typical of *E. coli*, and their presence in all the studied isolates reinforces the identification of these strains as *E. coli*.

Particular attention was given to the strains KNH8, KNH11, KNH14, and KNH16. Amplification, sequencing, and sequence alignment using the NCBI search engine confirmed that these strains belong to the genus *E. coli*. However, it should be noted that KNH8 (PQ86481) is closely related to ETEC, while KNH11 (PQ864812), KNH14 (PQ864813), and KNH16 (PQ864814) are related to EPEC.

### 3.2. In Vitro Evaluation of the Pathogenicity of the *E. coli* Genus

#### 3.2.1. Acid pH Resistance and Bile Salt Resistance Test

**Figure 1:**
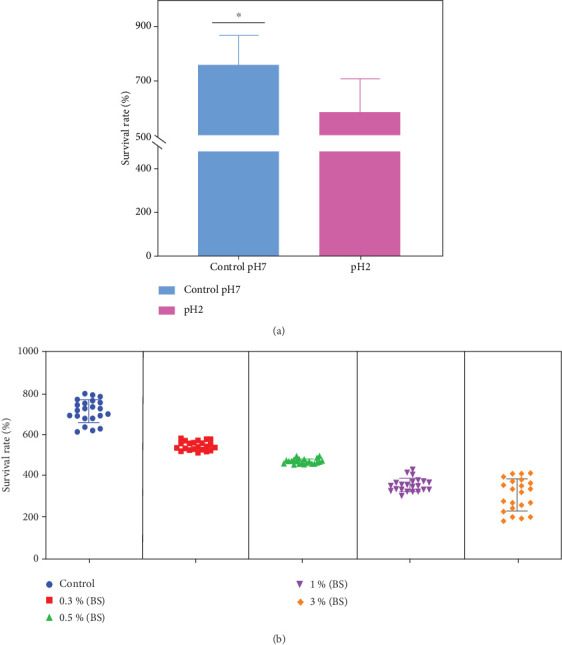
Curves showing the variation (a) in survival rates under acidic pH conditions (pH 2) and (b) in the presence of various bile salt concentrations. A total of 22 isolates were subjected to this test including KNH1, KNH2, KNH3, KNH4, KNH5, KNH6, KNH7, KNH8, KNH9, KNH10, KNH11, KNH12, KNH14, KNH16, KNH17, KNH18, KNH19, KNH20, KNH21, KNH22, KNH23, and KNH24. Sf- Spa40- and *S. flexneri* were used as control. BS, biosurfactant. ⁣^∗^ shows the standard deviation differences between two analytical conditions.

All tested isolates including KNH1, KNH2, KNH3, KNH4, KNH5, KNH6, KNH7, KNH8, KNH9, KNH10, KNH11, KNH12, KNH14, KNH16, KNH17, KNH18, KNH19, KNH20, KNH21, KNH22, KNH23, and KNH24 exhibited significant resistance to gastric pH. Sf-Spa40- and *Shigella flexneri* were used as controls (Figure 1). This observation was made by assessing the survival rate of the isolates, followed by culturing on agar. The results indicate that a survival rate greater than 100% signifies that bacterial growth was not inhibited; on the contrary, the bacteria continued to multiply despite the high acidity of the medium. Isolates cultured in broth at pH 2 not only survived but also continued to multiply almost as effectively as isolates cultured in a neutral medium, which served as a control.

All tested isolates demonstrated significant resistance to various concentrations of bile salts (0.3%, 0.5%, 1%, and 3%) (Figure 1b). This observation was made by evaluating the survival rate of the isolates at each concentration, followed by culturing on agar (Figure 1b). This means that the isolates not only survived the conditions imposed by the bile salts but also continued to multiply in these environments (Figure 1).

#### 3.2.2. Biofilm Formation, Detection of Swarming Ability, Hemolytic Activity, and Antibiogram Test

**Figure 2:**
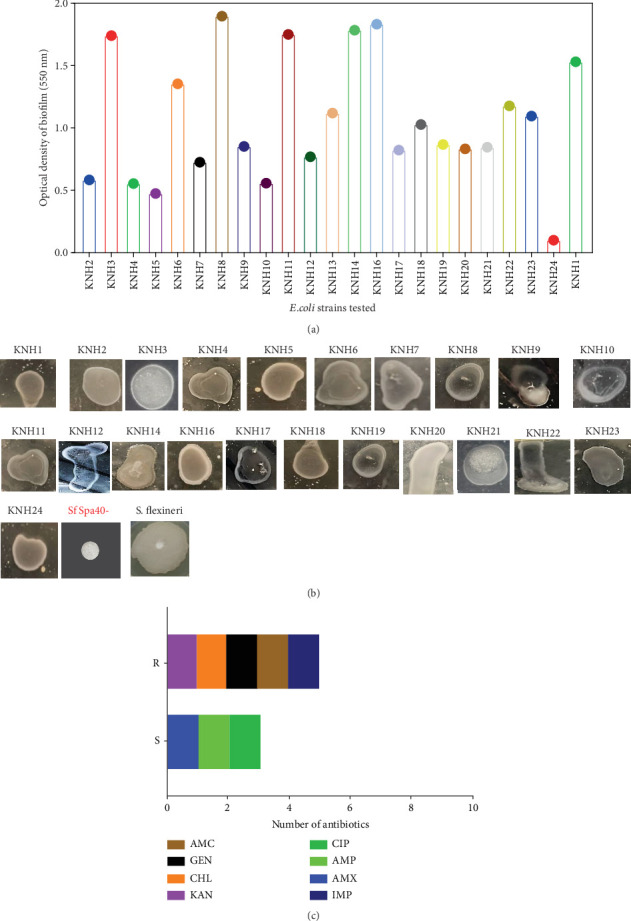
(a) Quantitative variation of biofilm formation. (b) Swarming tests of the isolates. (c) Antibiogram test. A total of 22 isolates were subjected to these tests, including KNH1, KNH2, KNH3, KNH4, KNH5, KNH6, KNH7, KNH8, KNH9, KNH10, KNH11, KNH12, KNH14, KNH16, KNH17, KNH18, KNH19, KNH20, KNH21, KNH22, KNH23, and KNH24. Sf Spa40- and *S. flexneri* have been used as controls.

Biofilm formation was evaluated using both qualitative and quantitative methods. Beyond an OD of 1, 45% of the bacteria form strong biofilms (KHN1, KNH3, KNH6, KNH8, KNH14, KNH16, KNH18, KNH22, KNH23, and KNH24). Between 0.5 and 0.8, 45% of intermediate biofilms are observed (KNH2, KNH4, KNH7, KNH9, KNH10, KNH12, KNH17, KNH19, KNH20, and KNH21). Below 0.5, 9% of the isolates form weak biofilms, including the strains (KHN5 and KHN24) (Figure 2a).

In terms of detection of swarming ability, all isolates, including KNH1, KNH2, KNH3, KNH4, KNH5, KNH6, KNH7, KNH8, KNH9, KNH10, KNH11, KNH12, KNH14, KNH16, KNH17, KNH18, KNH19, KNH20, KNH22, KNH23, and KNH24, were capable of swarming in semisolid medium. *Shigella* wild type and mutant strains were used as internal controls in this study. It is likely that differences in the intensity of swarming were observed (Figure 2b). The hemolysin test conducted on a total of twenty-two isolates revealed that ten isolates (45.45%) exhibited gamma hemolysis, characterized by the absence of complete erythrocyte lysis. Meanwhile, twelve isolates (54.54%) displayed alpha hemolysin production, indicating the absence of red blood cell lysis (data not shown).

Based on biofilms test, four strains have been chosen for the following tests (this included KNH8, KNH11, KNH14, and KNH16). An antibiogram test was conducted on a set of four bacteria selected based on their pathogenicity. Eight different antibiotics were used to evaluate the sensitivity of the isolates, namely, imipenem (IMP), amoxicillin (AMX), ampicillin (AMP), ciprofloxacin (CIP), kanamycin (KAN), chloramphenicol (CHL), gentamicin (GEN), and amoxicillin/clavulanic acid (AMC). The results show all *E. coli* strains demonstrate a high resistance to amikacin, KAN, CIP, GEN, and CHL. Meanwhile, three out of eight antibiotics including AMP, IMP, and AMX showed good activities on strains (Figure 2c).

### 3.3. In Vivo Evaluation of Pathogenicity of Four Strains (KNH8, KNH11, KNH14, and KNH16)

**Figure 3:**
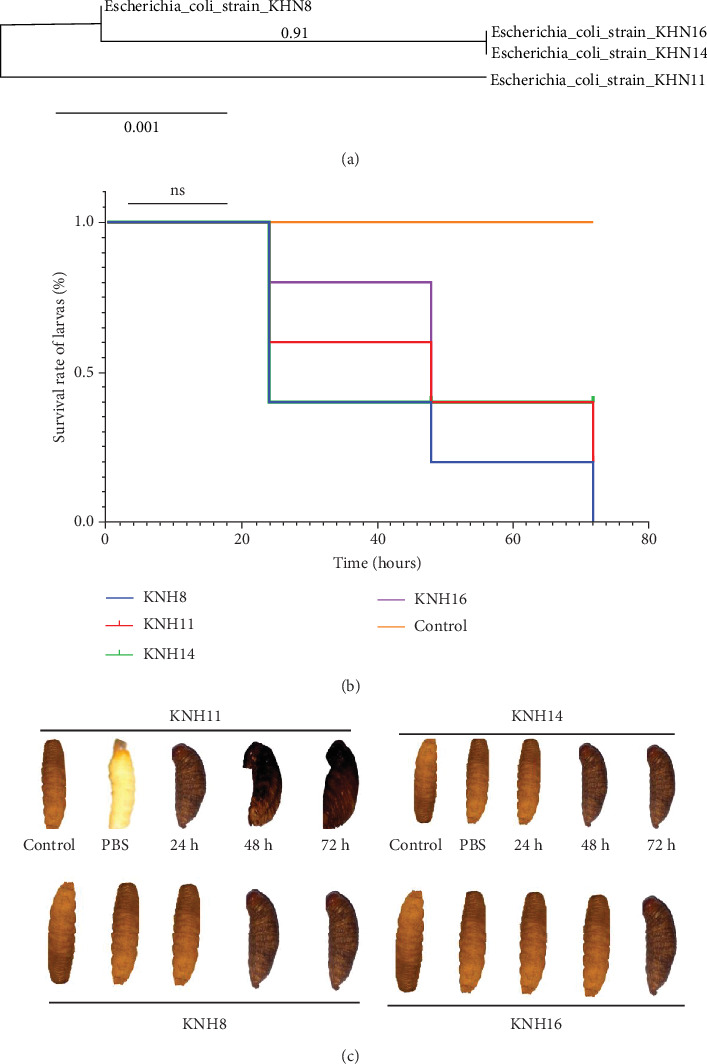
(a) Phylogenic tree showing the relationship of KNH8, KNH11, KNH14, and KNH16. (b) Larval survival rate over time with different suspected pathogenic isolates. (c) Immune response of larvae to the virulence of the tested strains, evidenced by melanization (blackening of the larvae). *E. coli* strains (KNH8, KNH11, KNH14, and KNH16).

We first identified the isolates KNH8, KNH11, KNH14, and KNH16 using 16S rRNA analysis. The phylogenetic tree demonstrating the relatedness of KNH8 (PQ86481), KNH11 (PQ864812), KNH14 (PQ864813), and KNH16 (PQ864814) to ETEC and EPEC has been constructed (Figure 3a).

The pathogenicity of *E. coli* strains (KNH8, KNH11, KNH14, and KNH16) was analyzed using the larvae of *R. phoenicis* (Nsombé). The results showed that after 72 h, the larvae exhibited a significant immune response to all tested strains, evidenced by the presence of melanization (Figure 3). The virulence varied from one strain to another. However, no immune response was observed in larvae inoculated with PBS or the control larvae (0.1X). The bacterial isolate KNH11 demonstrated high pathogenicity, with a mortality rate of 67% after 24 h and 100% after 48 h. The isolates KNH8 and KNH14 did not cause larval death after 24 h but showed 100% mortality at 48 h. On the other hand, the KNH16 isolate displayed a slightly slower kinetics, with a mortality rate of 33% at 24 h and 100% at 72 h. The survival rate of the larvae after the experiment is shown. The following figure presents the results of the immune response based on the virulence of the strains (Figure 3,c). The melanization aspects also indicate that the KNH11 strain appears to be the most virulent (Figure 3).

### 3.4. Impact of Rose Bengal on Pathogenic Mechanisms in *E. coli*

#### 3.4.1. Immune Response of Larvae to Bacteria Pretreated With Rose Bengal and Gastric pH Resistance Test in the Presence of Rose Bengal

**Figure 4:**
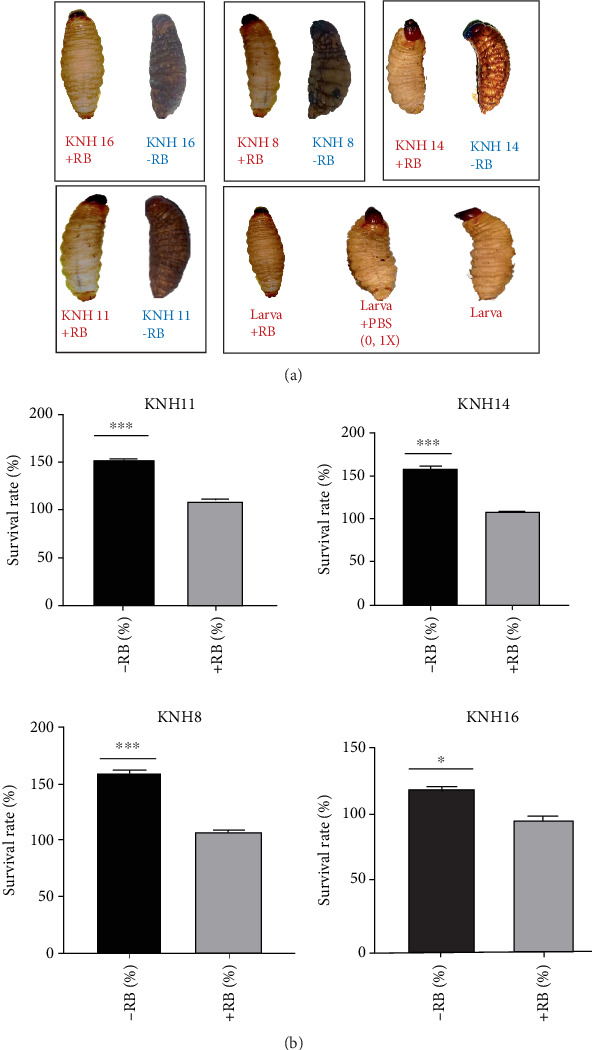
(a) Immune response of larvae to bacteria pretreated with Rose Bengal. (b) Comparison of survival rates at acidic pH 2 in the presence and absence of Rose Bengal of *E. coli* strains (KNH8, KNH11, KNH14, and KNH16). ⁣^∗^ indicates significant difference. ⁣^∗∗∗^ indicates highly significant difference.

The four strains were pretreated with Rose Bengal for 24 h before being inoculated into the larvae. The results show that the strains treated with Rose Bengal did not trigger an immune response in the larvae, in contrast to the immune activation observed following the injection of untreated bacteria (Figure 4a). A similar absence of immune response was observed with the injection of 0.1 M PBS, which was used as a control (Figure 4a).

To assess the impact, a range of Rose Bengal concentrations was prepared: 500, 400, 300, 200, 100, 75, 50, 25, 15, and 2.5 *μ*M. The IC50 of 200 *μ*M was selected for evaluating pathogenicity of *E. coli* strains (KNH8, KNH11, KNH14, and KNH16). This concentration represents the minimum inhibitory concentration of Rose Bengal's biological functions in *E. coli* in this study. The pathogenicity test at gastric pH, conducted with a 200 *μ*M concentration of Rose Bengal, showed that after 24 h, the survival rate of bacteria in an acidic medium (pH 2) containing Rose Bengal was significantly reduced (*p* < 0.05) and considerably lower than that of bacteria cultured in an acidic medium without Rose Bengal. The survival rate of bacteria in the medium without Rose Bengal reached approximately 700%, while in the medium with Rose Bengal, it did not exceed 100%, as illustrated in Figure 4b.

#### 3.4.2. Bile Salt Resistance Test in the Presence of Rose Bengal

**Figure 5:**
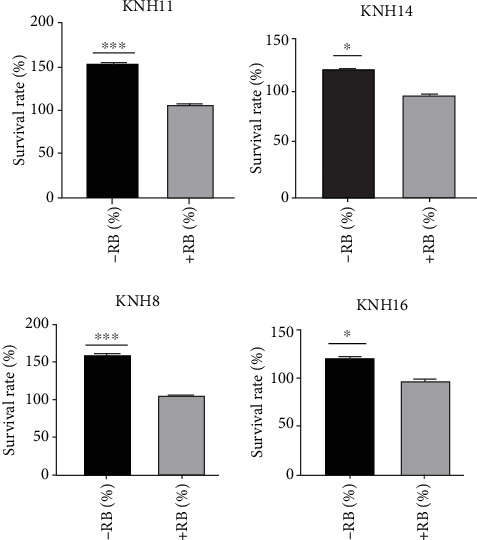
Survival rate in the presence and absence of Rose Bengal in the bile salts of *E. coli* strains (KNH8, KNH11, KNH14, and KNH16). ⁣^∗^ indicates significant difference. ⁣^∗∗∗^ indicates highly significant difference.

The pathogenicity test in the presence of bile salts, conducted with a 200 *μ*M concentration of Rose Bengal, revealed that after 24 h, the survival rate of bacteria in the medium was significantly lower (*p* < 0.05) than that observed in the medium without Rose Bengal. Indeed, the survival rate in the medium without Rose Bengal reached 600%, while it did not exceed 100% in the medium with Rose Bengal, as illustrated in Figure 5.

#### 3.4.3. Biofilm Formation and Hemolytic Activity in the Presence of Rose Bengal

**Figure 6:**
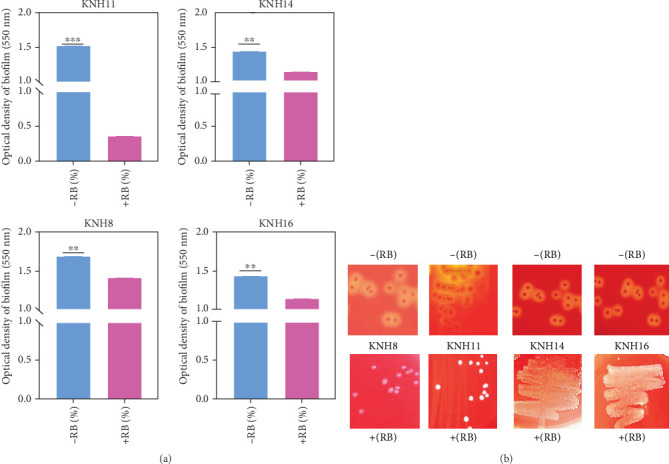
(a) Variation in OD of biofilms formed in the presence and in the absence of Rose Bengal of E. coli strains (KNH8, KNH11, KNH14, and KNH16). (b) Hemolytic activity of *E. coli* strains (KNH8, KNH11, KNH14, and KNH16). ⁣^∗∗^ indicates moderate significant difference. ⁣^∗∗∗^ indicates highly significant difference.

The biofilm formation test in the presence of Rose Bengal revealed that Rose Bengal had a significant impact on biofilm formation. Specifically, the bacteria cultivated with Rose Bengal exhibited a significantly lower (*p* < 0.05) biofilm formation rate compared to those cultivated without Rose Bengal (Figure 6). The analysis of hemolytic activity of bacterial isolates in the presence of Rose Bengal was conducted to better understand the impact of Rose Bengal compound on their hemolytic profile. In the absence of Rose Bengal, the strains KNH8, KNH11, KNH14, and KNH16 exhibited an alpha-hemolysis phenotype (Figure 6). In the presence of Rose Bengal, an inhibition of hemolysin production was observed, resulting in the absence of hemolysis, corresponding to gamma-hemolysis (Figure 6).

#### 3.4.4. Swarming Motility in the Presence of Rose Bengal

**Figure 7:**
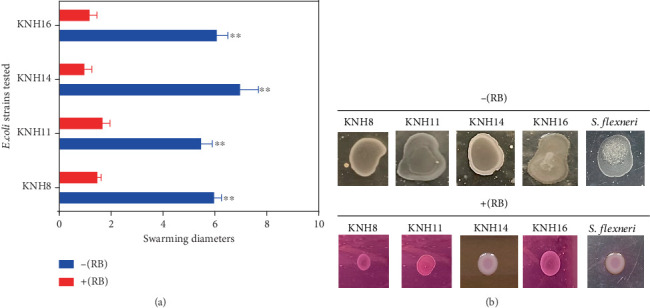
(a, b) Swarming of *E. coli* strains (KNH8, KNH11, KNH14, and KNH16) in the presence and in the absence of Rose Bengal (−[RB]) and in the presence of Rose Bengal (+[RB]). ⁣^∗∗^ indicates moderate significant difference.

The results show that bacteria in the presence of Rose Bengal swarm very weakly, with a very small swarming diameter (*p* < 0.05) compared to those cultured without Rose Bengal (Figure 7). Specifically, the swarming diameters of bacteria cultivated without Rose Bengal were significantly larger, ranging from 6 to 8 mm, while those of bacteria cultured with Rose Bengal were all smaller than 2 mm (Figure 7a,b).

#### 3.4.5. Antibiotic Resistance of Strains in the Presence of Rose Bengal

**Figure 8:**
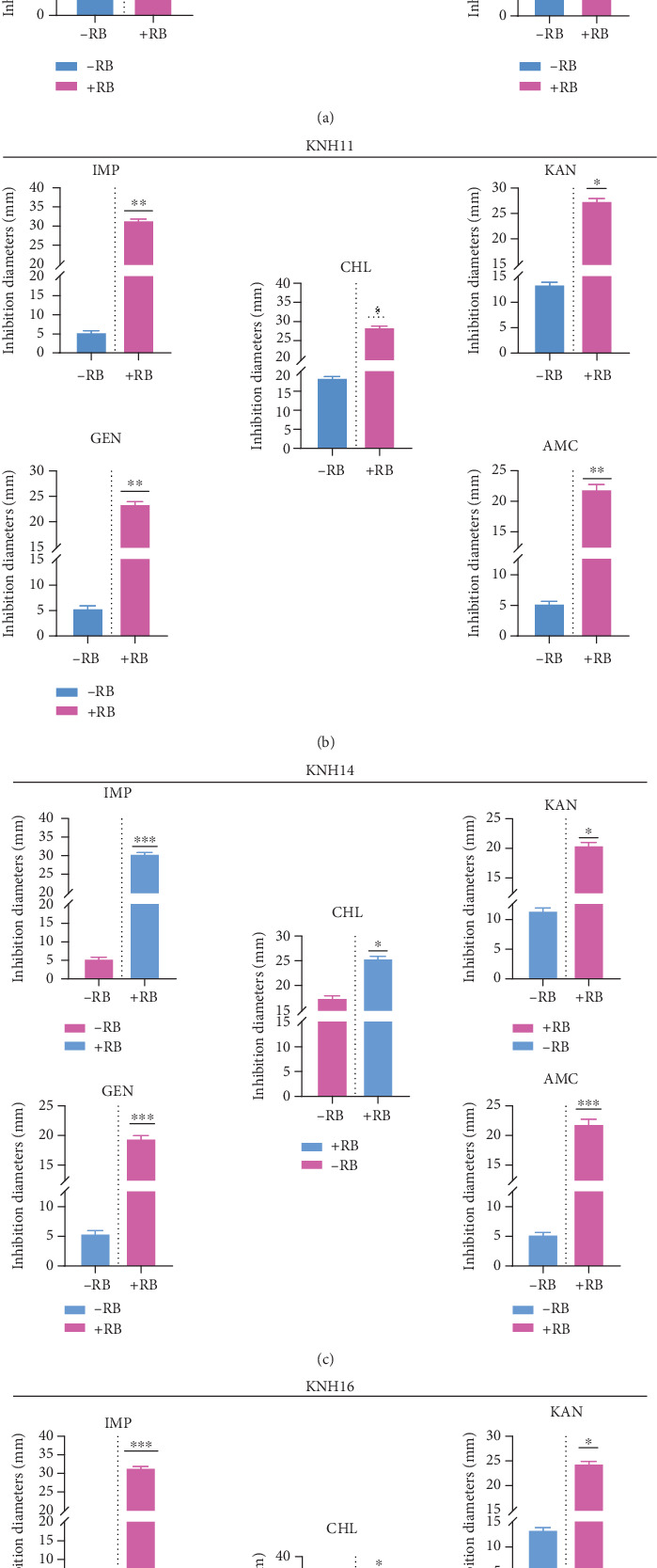
Amplification of diameters of antibiotics in the presence of Rose Bengal. *E. coli* strains (a) KNH8, (b) KNH11, (c) KNH14, and (d) KNH16 in the presence and in the absence of Rose Bengal (−[RB]) and in the presence of Rose Bengal (+[RB]). Imipenem (IMP), kanamycin (KAN), chloramphenicol (CHL), gentamicin (GEN), and amoxicillin/clavulanic acid (AMC). ⁣^∗^ indicates significant difference. ∗∗ indicates moderate significant difference. ⁣^∗∗∗^ indicates highly significant difference.

Four strains were selected to evaluate their sensitivity to various antibiotics, including IMP, KAN, CHL, GEN, and AMC. In the presence of Rose Bengal, the sensitivity of the KHN8 strain to IMP increased fivefold, to KAN twofold, to CHL twofold, to GEN fourfold, and to AMC fivefold (Figure 8a). For the KHN11 strain, the sensitivity to IMP increased sixfold, to KAN twofold, to CHL twofold, to GEN fivefold, and to AMC fourfold (Figure 8b). Finally, for the KHN14 and KHN16 strains, the sensitivity was similar with to IMP increased sixfold, to KAN twofold, to CHL 1.6-fold, to GEN fourfold, and to AMC fourfold (Figure 8c,d).

## 4. Discussion

This study highlights the promising therapeutic potential of Rose Bengal against *E. coli* infections, targeting several mechanisms related to pathogenicity and bacterial resistance to antibiotics. Pathogenic strains of *E. coli* circulate within the Congolese population.

By inhibiting the SecYEG system [10], Rose Bengal reduces bacterial virulence expression by preventing biofilm formation, swarming motility, and weakening resistance to digestive tract barriers. Additionally, it enhances *E. coli* sensitivity to antibiotics such as AMX, AMP, and CIP, underscoring its potential role as an adjunct in antibacterial therapies. The finding of this work paves the way for future research, including in vivo and clinical studies, to assess the safety and effectiveness of Rose Bengal in more complex models. The development of combined treatments, integrating Rose Bengal to enhance the action of existing antibiotics, could offer new solutions to combat resistant infections. Investigating the resistance mechanisms not affected by Rose Bengal, as well as developing new formulations such as gels or antimicrobial coatings, presents promising avenues to extend its applications in medical and industrial fields [15] .

Our findings pointed out that *E. coli* strains including KNH8, KNH11, KNH14, and KNH16 form stable biofilms at 37°C. Additionally, the same strains can exhibit significant motility. This motility is a mechanism allowing them to move across solid surfaces while forming extended colonies, enabling them to colonize various environments rich in substrates necessary for bacterial growth and also to escape certain predators [1, 16–19]. All strains (KNH8, KNH11, KNH14, and KNH16) can grow and multiply at pH levels close to those found in the stomach (pH 2) and can survive extreme conditions in the presence of high bile salt concentrations [20, 21]. This suggests that these *E. coli* strains can cross the barriers formed by bile and gastric secretions in the stomach to actively colonize the colon and manifest their virulence. The ability of these bacteria to survive in these hostile environments has already been reported in the literature [22]. Furthermore, these bacteria are capable of strongly adhering to tissues by forming an interactive cellular biofilm network [23, 24].

Our study also demonstrated the multidrug resistance of the *E. coli* strains (KNH8, KNH11, KNH14, and KNH16). This characteristic of multiresistance to locally prescribed antibiotics poses a real threat to the management of patients suffering from these infections [25]. All these pathogenic aspects prompted us to investigate the synergy between Rose Bengal and antibiotics in the search for a palliative solution.

The larvae of *R. phoenicis* (Nsombé) were used in this study as an animal model to evaluate the pathogenicity of the *E. coli* strains. The melanization would indicate the virulence of the tested strains. It is noteworthy that this virulence varied from one bacterium to another in terms of its ability to induce melanization and cause larval death [26]. These results suggest that the larvae constitute a good animal model to evaluate the pathogenicity of bacteria involved in diarrhea. Moreover, these *E. coli* strains are highly pathogenic, capable of crossing the barriers of acidity and bile salts to reach the colon and secrete virulence effectors, thereby causing diarrhea.

The effect of Rose Bengal on *E. coli* was assessed in this study, and an IC50 of 200 *μ*M was determined. The results show that Rose Bengal significantly decreases the resistance of bacteria to gastric acidity and bile salts. After 24 h, the survival rate decreased from 700% to 200% in the acidic medium, from 600% to 100% in the bile salt–containing medium, and from 700% to 100% in the pH 2 medium. The mechanism of action of Rose Bengal is based on its ability to inhibit the SecA protein, which is essential to the bacterial secretion system SecYEG [10]. The SecA protein facilitates the translocation of proteins to bacterial membranes, some of which are crucial for resistance to acidic pH and bile salts. Thus, the inhibition of SecA blocks the translocation of proteins such as CadA/CadB and GadA/GadB, which are involved in pH regulation, or TolC, OmpF, and OmpC, which are responsible for bile salt export, thereby reducing bacterial survival in these hostile environments [21, 27]. Previous studies have shown another potential of Rose Bengal as an antimicrobial agent, particularly in acidic environments [28]. Rose Bengal significantly reduces bacterial survival in an acidic environment by increasing their permeability and causing destabilization through intense oxidative stress, which damages the membranes, proteins, and bacterial DNA [29, 30]. In acidic conditions, bacteria activate adaptive mechanisms, such as proton pumps, to regulate their internal pH. However, Rose Bengal inhibits these mechanisms by oxidizing critical enzymes involved in acid resistance, thereby exacerbating bacterial adaptation difficulties.

*E. coli* is a bacterium capable of secreting several proteins, including hemolysins, which play a key role in host cell lysis. In addition to hemolysins, other virulent factors, such as toxins and adhesins, also contribute to the pathogenicity of this bacterium [31, 32]. These proteins are transported by various secretion systems, among which the Sec system plays a central role by facilitating the translocation of proteins across the bacterial plasma membrane [33].

The inhibition of the Sec system by Rose Bengal blocks the effective secretion of hemolysins and other virulence factors, reducing their availability to interact with target cells and cause their lysis. This mechanism could explain the decrease in hemolytic activity observed during experimental tests. By limiting *E. coli*'s ability to release these proteins, Rose Bengal directly affects its potential to induce cellular damage, thereby highlighting the importance of the Sec system in bacterial pathogenicity [34].

Meanwhile, tests on biofilm formation revealed that Rose Bengal significantly reduces this ability in *E. coli* isolates. Biofilm formation, a crucial characteristic of pathogenicity, is affected by the inhibition of SecA, a protein essential for the SecYEG secretion system. Adhesion proteins such as FimH and PapG, which are necessary for bacterial cell attachment to surfaces, as well as components of the extracellular matrix, such as exopolysaccharides produced by PgaABCD, rely on this system for their export [35]. By inhibiting SecA, Rose Bengal reduces the bacteria's ability to form robust biofilms, explaining the low ODs measured during the tests, in agreement with previous studies [36]. The OD of the biofilms, measured at 550 nm, confirmed this inhibitory effect. The alteration of the extracellular matrix by reactive oxygen species (ROS) generated by Rose Bengal appears to be the main cause of this reduction. In addition, a decrease in motility was observed, with swarming diameters reduced to less than 2 mm in the presence of Rose Bengal. This collective behavior, which facilitates colonization, depends on flagella whose biogenesis relies on proteins such as FlhA, FlhB, and FliC exported by SecA. The inhibition of SecA by Rose Bengal directly disrupts these flagella-related signaling systems, thereby compromising the production of functional flagella [37]. These results confirm the impact of Rose Bengal on reducing mechanisms associated with bacterial virulence, particularly through the significant decrease in swarming motility [36].

These bacteria are resistant to commonly used antibiotics in the Republic of Congo and can form biofilms, produce hemolysins, and bypass barriers in the digestive tract [1]. The antibiogram revealed a notable increase, reaching up to 100%, in sensitivity to certain antibiotics initially associated with resistance. These observations reflect a complex interaction between the inhibition of SecA by Rose Bengal and bacterial resistance mechanisms [10]. Previous studies demonstrated that photosensitizers, such as Rose Bengal, alter bacterial membrane permeability. This phenomenon facilitates the entry of antibiotic molecules by disrupting cellular barriers. Specifically, the modifications of membrane structures, including porins (OmpF and OmpC), induced by Rose Bengal, play a key role in enhancing the efficacy of *β*-lactam antibiotics such as AMX and AMP. Additionally, Rose Bengal appears to inhibit *β*-lactamases, thereby reducing the bacteria's ability to hydrolyze these antibiotics, which strengthens their activity [36]. Regarding aminoglycosides, such as KAN and GEN, which require active transport to cross the membrane, the membrane disruption induced by Rose Bengal can significantly enhance their penetration. This increase in membrane permeability facilitates the active transport required by these antibiotics, increasing their intracellular concentration and, consequently, their efficacy [38]. The inhibition of efflux pumps, particularly AcrAB-TolC, also contributes to this increased sensitivity by limiting the expulsion of antibiotics and increasing their intracellular concentration [39]. However, it is important to note that alternative systems such as Tat and Lol, which are not SecA-dependent, remain functional, allowing residual secretion of certain essential proteins. This explains why the effect of Rose Bengal is not completely lethal. Furthermore, the results show that the efficacy of Rose Bengal is concentration-dependent, and future studies should explore its synergy with inhibitors targeting other secretion pathways to maximize its antimicrobial effectiveness. Moreover, it could be important to study the cytotoxicity on various human cell lines while considering different concentrations of Rose Bengal. Several studies have demonstrated that Rose Bengal has been used in anticancer therapy [40–43], and many other studies clearly show the nontoxicity of Rose Bengal alone or in combination with molecules in eukaryotic cells including human corneal epithelial cell and fibroblast [40]. Rose Bengal photodynamic antimicrobial therapy (RB-PDAT) has been used as a potential treatment for progressive infectious keratitis. Human cells showed no apparent cytotoxicity. RB-PDAT is safe on the corneal endothelium and has no effect on the limbal stem cell viability or function [44].

We are aware that this research is fundamental to addressing the issue of antibiotic resistance. Many other studies are currently being carried out in our laboratory, including the impact of Rose Bengal on gut microbiota and host immune responses, as well as the synergy between Rose Bengal and other antimicrobial agents, particularly those targeting non-SecA-dependent pathways.

We are planning to conduct a collaboration with other laboratories in the field of virology, particularly by studying the potential of Rose Bengal combined with phage and developing and testing novel formulations of Rose Bengal, such as nanoparticles, gels, or coatings, to enhance its stability, delivery, and efficacy. All aforementioned studies will allow us to design and conduct clinical trials to assess the safety, tolerability, and efficacy of Rose Bengal in human patients with *E. coli* infections.

## 5. Conclusion

This study underscores the therapeutic potential of Rose Bengal as a promising agent against *E. coli* infections, targeting multiple mechanisms associated with pathogenicity and antibiotic resistance. Pathogenic *E. coli* strains are prevalent within the Congolese population, displaying resistance to commonly used antibiotics and exhibiting virulence traits such as biofilm formation, hemolysin production, and the ability to overcome digestive tract barriers.

Rose Bengal demonstrates significant antibacterial properties by inhibiting the SecYEG translocon, thereby reducing bacterial virulence through the suppression of biofilm formation, swarming motility, and resistance to digestive tract barriers. Furthermore, it enhances the sensitivity of *E. coli* to several antibiotics, including KAN, AMX, AMP, and CIP, highlighting its potential as an adjuvant in antimicrobial therapies.

The findings of this study provide a strong foundation for further research, including in vivo and clinical trials, to evaluate the safety and efficacy of Rose Bengal in complex biological systems. Developing combination therapies that incorporate precise concentrations of Rose Bengal to potentiate the effectiveness of existing antibiotics could offer innovative strategies to address resistant infections. Additionally, investigating resistance mechanisms unaffected by Rose Bengal and exploring advanced delivery systems, such as gels or antimicrobial coatings, represent promising directions for expanding its applications in both medical and industrial contexts.

## Data Availability

The data that support the findings of this study are available from the corresponding author upon reasonable request.
